# Agrivoltaic Impact on Some Lettuce Quality Attributes and Photovoltaic Power Generation

**DOI:** 10.3390/plants14182853

**Published:** 2025-09-12

**Authors:** Yasin Gunhan, Onur Taskin

**Affiliations:** 1 Biosystems Engineering Department, Institute of Natural and Applied Science, Bursa Uludag University, 16059 Bursa, Türkiye; 2 Bursa Directorate of Provincial Agriculture and Forestry, 16170 Bursa, Türkiye; 3 Biosystems Engineering Department, Faculty of Agriculture, Bursa Uludag University, 16059 Bursa, Türkiye

**Keywords:** dual land use, photovoltaic panels, light intensity, SPAD, color, irrigation

## Abstract

Agrivoltaics represent an innovative approach that enables the simultaneous production of both agriculture and energy on limited lands. The purpose of this study is to evaluate the feasibility of using pruning residues as a sustainable construction material for Agrivoltaic structures and to investigate the different irrigation conditions (control group (open field) with 100% irrigation (I), 60% I, 80% I, 100% I, and 120% I under the Agrivoltaic) on some lettuce (*Lactuca sativa* L.) quality parameters and PV (photovoltaic) power generation. As a result of the study, the Agrivoltaic system resulted in a 2.92% power loss compared to a conventional PV panel. The average light intensity under the PV panels was recorded as 6500 lux, while it was 47,700 lux in the control group. Therefore, the mean SPAD values of lettuce plants were found at 24.94 SPAD under the Agrivoltaic system, in contrast to 32.49 SPAD measured in the control group. Among the conditions tested, the 100% irrigation (I) condition under the Agrivoltaic system was found to be the most favorable method for lettuce quality in terms of the a* color value, head weight, marketable head weight, head height, head diameter, root length, root width, and root collar diameter. In conclusion, Agrivoltaic systems demonstrate significant potential in the agricultural and energy sectors, contributing to environmental and economic sustainability.

## 1. Introduction

The installation of solar power plants on limited land surfaces has led to competition with agricultural production. Therefore, integrated systems known as agrivoltaics have been developed. Although Agrivoltaic systems improve the efficiency of land utilization and contribute to environmental sustainability by reducing the fossil fuel consumption of agricultural production, there is concern that insufficient exposure to sunlight may negatively impact crop yield and quality in regions with limited solar radiation [1,2].

In the literature, various Agrivoltaic designs have been studied in conjunction with different crops. Although alternative types such as vertical panels have been applied, the most common design consists of fixed panels mounted above crops [3]. However, these systems require a greater amount of construction materials compared to conventional solar power plants. Previous studies have investigated various crops in yields, including tomatoes (200% increase) and hot peppers (300% increase) in the USA, lettuce (1–58% decrease) in France, alfalfa (5–8% decrease) in Germany, and corn (4.3–12.5% increase) in Italy [4].

Lettuce (*Lactuca sativa* L.) is a considerable vegetable due to its high nutritional value and numerous health benefits. Beyond its antioxidant capacity, it contains substantial amounts of folate and dietary fiber. This composition helps support heart health, lower cholesterol levels, and combat cancer. It also promotes the regular functioning of the digestive system and aids in weight management [5]. Therefore, lettuce consumption is widespread worldwide, and the leading countries in lettuce production (in tons) in 2022 were China (14,982,813), the USA (3,298,933), India (1,161,251), Spain (969,190), and Italy (638,180) according to FAO data [6].

Lettuce can be cultivated throughout the year using both protected cultivation techniques and open-field agriculture. Cultivation in Agrivoltaic systems is expected to enhance production efficiency, as the crop can grow successfully under low radiation conditions [7]. In the literature for lettuce production under various Agrivoltaic designs, Carreño-Ortega et al. [8] assessed the feasibility of using simultaneously, by different shades of photovoltaic panels (rooftop Agrivoltaic) and by lettuce crops in terms of fresh weight, biomass production values, number of leaves, the maximum length of leaves, and humidity. An increase in land productivity was quantified by Cossu et al. [9] following the integration of an experimental vertical farm for baby leaf lettuce into a pre-existing commercial closed Agrivoltaic system. On the other hand, Elamri et al. [10] conducted model simulations aimed at improving both land use efficiency and water productivity in an Agrivoltaic system situated in Lavalette, Montpellier, France.

The central hypothesis of this research is that an Agrivoltaic system constructed from pruning residues can provide sufficient structural integrity and an environmentally sustainable alternative to conventional steel mounting structures for PV panel installation while simultaneously creating a favorable microclimate that supports lettuce growth, particularly when irrigation is properly managed. The first objective of the research was to compare the power performance of PV panels mounted on an Agrivoltaic design with that of those installed on conventional ground-mounted systems. In addition, the study evaluates the effects of different irrigation levels (60% I, 80% I, 100% I, and 120% I) on lettuce growth under the Agrivoltaic system and determines plant responses in terms of weight, number of leaves, plant and root sizes, color, leaf temperature, and SPAD values.

## 2. Results and Discussion

### 2.1. Climatic Conditions During the Experimental Period

**Figure 1:**
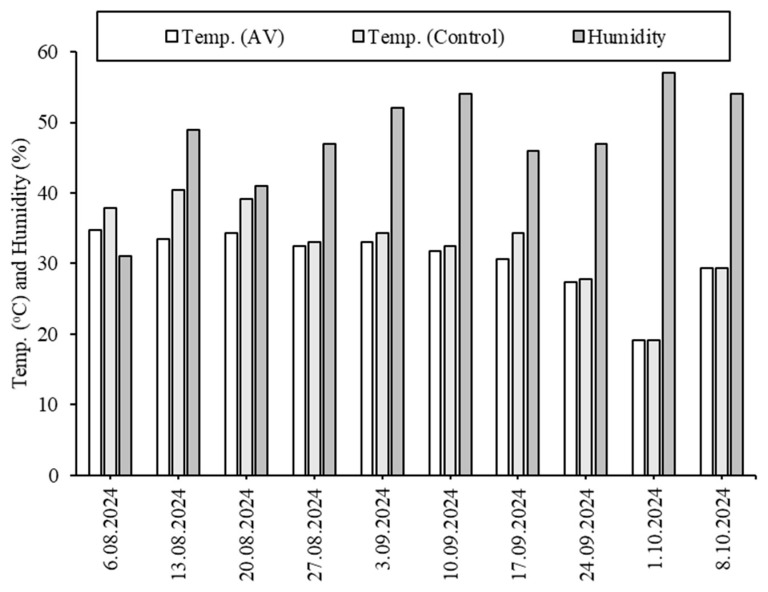
Daily variations in climatic conditions during the experimental period.

Figure 1 presents the climatic data recorded during the study period, including solar radiation, temperature, humidity, and wind speed direction. The highest temperature recorded was 40.4 °C in the control group on 6 August 2024, while the lowest temperature was 19.1 °C under the PV panels on 1 October 2024. Overall, temperatures under the PV panels remained lower than those in the control group regarding the shading effect. Humidity levels increased over time, rising from 31% on 6 August 2024 to 54% on 8 October 2024, with the highest recorded humidity of 57% on 1 October 2024. Wind speeds remained low (1.62 m/s), with the highest wind speed of 3 m/s recorded on 3 September 2024. The predominant wind direction was determined to be Southwest–Northeast (SW–NE). The solar radiation values ranged between 908 and 180 W/m^2^ with an average of 743 W/m^2^.

**Figure 2:**
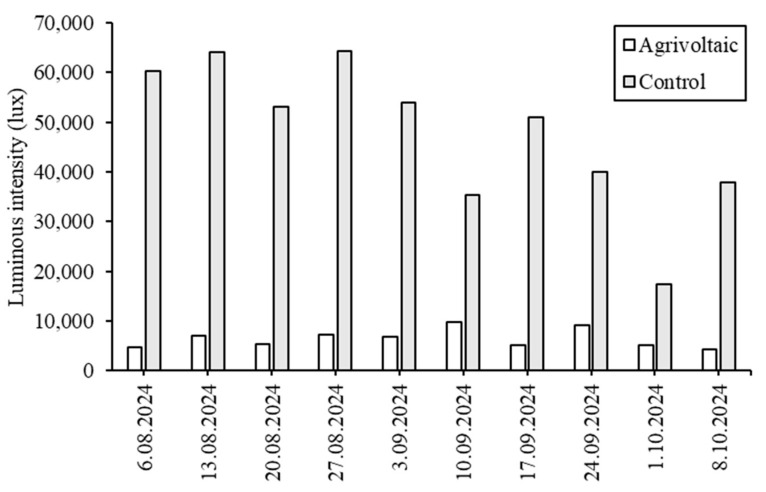
Comparison of light intensity recorded under the Agrivoltaic condition and in the control (open field).

The light intensity data were recorded during the study period, comparing the levels under the PV panels with those in the control group (Figure 2). The highest light intensity under the PV panels was measured at 9760 lux on 10 September 2024, while the control group recorded its highest level at 64,000 lux on 13 August 2024. The most significant difference between the two conditions was observed on 27 August 2024, marking a difference of 57,000 lux. Similarly, Ko et al. [11] reported that light intensity fluctuates continuously in Agrivoltaic setups and that the average light intensity is lower than in open-field conditions. The altered light conditions led to microenvironmental changes, primarily a 41% reduction in light. Else, Patel, and Chauhan [12] placed 12 panels per row (each with a 150 W output) in a 153.88 m^2^ area, all oriented southward. Their experimental study with a total of 48 panels found that both average and maximum light intensity levels were higher in open-field conditions compared to those under PV panels.

### 2.2. Leaf Temperature, SPAD, and Panel Power Under Agrivoltaic and Control Conditions

**Figure 3:**
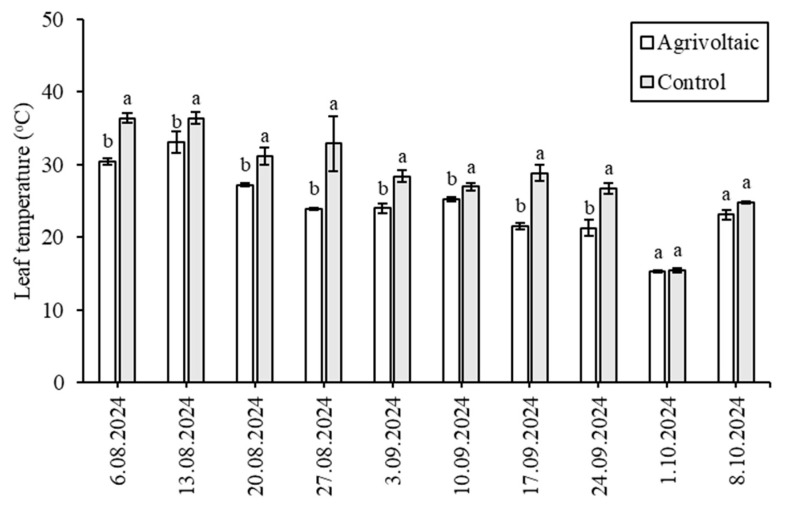
Leaf temperature measurements of lettuce plants under Agrivoltaic conditions and in the control (open field). The different letter indicates a significant difference (*p* < 0.05).

The leaf temperature of lettuce plants under PV panels compared to those in the control group is shown in Figure 3. The most significant difference between the two conditions was recorded on 27 August 2024, when the temperature under the PV panels was 23.90 °C, whereas in the control group, it was 32.87 °C (*p* < 0.05). Regarding the lowest recorded temperatures on 1 October 2024, the minimum temperature under the PV panels was 15.23 °C, while in the control group, it was slightly higher at 15.47 °C. Although lettuce plants under the PV panels exhibited lower temperatures due to the shading effect, no statistical difference was observed in the last two measurement days (*p* < 0.05). In previous studies, Disciglio et al. [13] conducted a study on medicinal plants grown in a dynamic Agrivoltaic system, comparing those in constant shade and partial shade with a control group. They found that plants under continuous shade consistently exhibited the lowest temperatures, ranging from 28.1 °C to 32.2 °C. Partially shaded plants showed slightly higher temperatures (30.0 °C to 35.1 °C) but were still lower than the control group (30.1 °C to 38.9 °C). Alternatively, Oleskewicz [14] investigated Agrivoltaic systems using experimental plots with panel gap distances of 2 ft, 3 ft, 4 ft, and 5 ft and a control plot with no panels, to assess their effects on pepper leaf temperature. The study reported average leaf temperatures of 67 °F, 66 °F, 66 °F, 66 °F, and 75 °F, respectively. Overall, leaf temperatures were lower under panel shading; however, no consistent trend was observed between increasing gap distance and leaf temperature.

**Figure 4:**
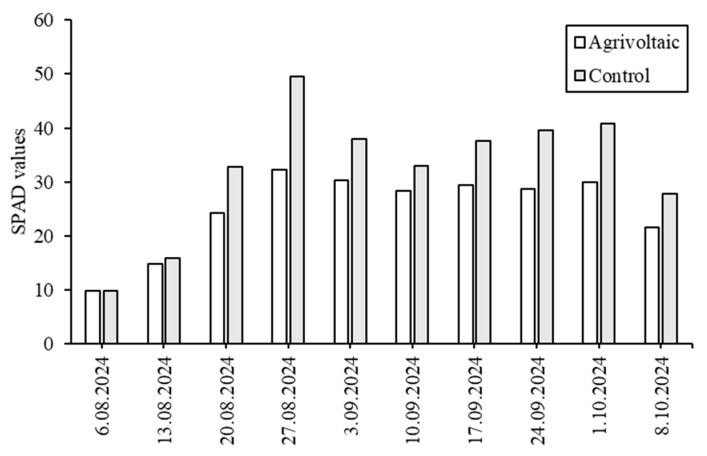
SPAD values of lettuce leaves grown under Agrivoltaic conditions and control conditions (open field).

The SPAD values were found to be lower in lettuce plants under the PV panels (Figure 4). The highest SPAD value under the PV panels was recorded at 29.95 SPAD on 1 October 2024, while the highest level in the control group was measured at 40.9 SPAD on 24 September 2024. Higher SPAD values generally indicate a greater chlorophyll concentration, which can enhance the plant’s ability to absorb light and perform photosynthesis efficiently. However, the factors of leaf age, nutrient availability, water stress, and environmental conditions can also influence both SPAD readings and the actual rate of photosynthesis. In contrast to our study, Potenza et al. [15] conducted research on soybeans in Italy and found that the SPAD value in the control group (43.58) was statistically higher than the value measured under the Agrivoltaic (AV2) system with 16% shading (41.87). However, three other shading treatments (27%, 9%, and 18%) showed no significant effect on SPAD values compared to the control group, with records of AV1 = 43.41, AV3 = 42.87, and AV4 = 42.33. Liu et al. [16] reported that although photosynthesis increases with rising light intensity, it plateaus once a saturation point is reached, beyond which photosynthetic efficiency may decline due to light saturation. This highlights that while sunlight is essential for healthy plant growth, excessive solar radiation can be detrimental. Accordingly, the use of Agrivoltaic systems may help mitigate the adverse effects of excessive solar exposure on crops.

**Figure 5:**
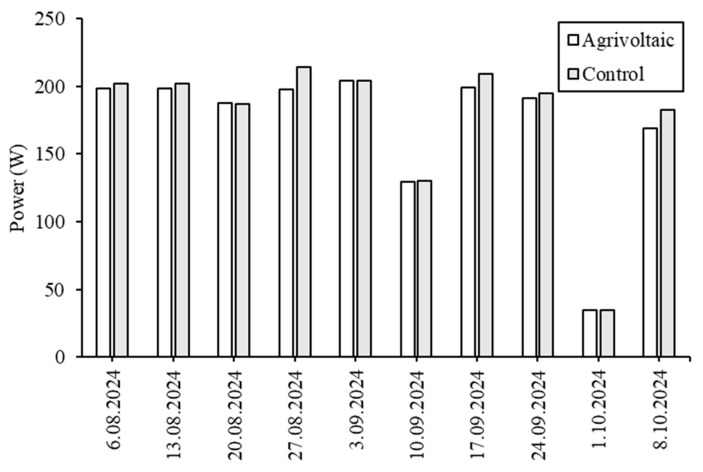
Power output comparison between the Agrivoltaic and ground-mounted PV panel (control).

Figure 5 illustrates that the PV panel on the ground-mounted generally exhibited higher power generation levels. On 27 August 2024, the power output of the ground-mounted PV panel was recorded at 214.1 W, while the Agrivoltaic PV panels produced 197.3 W, resulting in a difference of 16.8 W. In a study conducted in Germany, Trommsdorff et al. [17] reported that the electrical efficiency of an Agrivoltaic system—featuring a 5-m vertical clearance and a 19-m span—was approximately 17% lower than that of the reference system. Modi and Patel [18] compared the energy production of PV panels installed at heights of 0.91 m, 1.83 m, and 3.2 m, concluding that the 0.91 m height was the most optimal, generating a total of 6757 kWh during the study period. The variation in results reported in the literature may be attributed to factors such as panel heating due to grid connection, as well as design or climatic differences.

### 2.3. Size, Weight, and Number of Leaf Parameters of Lettuce

**Figure 6:**
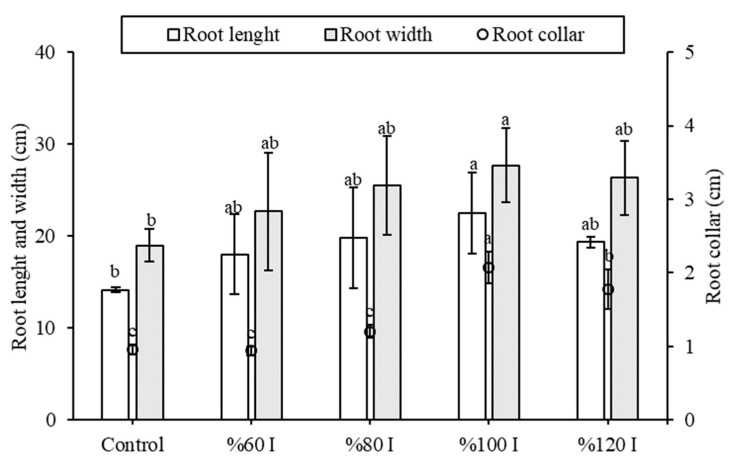
Root parameters of lettuce plants grown under different irrigation treatments in the Agrivoltaic and the control (open field). The different letter indicates a significant difference (*p* < 0.05).

A comparison of root length, root width, and root collar diameter among the control group and the %60 I, %80 I, %100 I, and %120 I groups under Agrivoltaic is presented in Figure 6. However, no significant differences in those values were found among %60 I, %80 I, %100 I, and %120 I (*p* < 0.05); the %100 I group under Agrivoltaic conditions exhibited the highest mean root length (22.50 ± 4.44 cm), mean root width (27.67 ± 4.04 mm), and root collar diameter (2.08 ± 0.21 mm). In contrast, the corresponding values in the control group were recorded as 14.17 ± 0.29 cm, 19.00 ± 1.58 mm, and 0.96 ± 0.06 mm, respectively. Additionally, the control group exhibited lower results compared to all Agrivoltaic conditions. Instead of full direct sunlight with high intensity at midday (open field-control), Agrivoltaic may reduce light stress on plants with a shading effect, and lower transpiration may reduce extreme fluctuations in water uptake demand. Therefore, the soil/root-zone temperature may moderate, supporting more consistent carbohydrate allocation to roots. Similarly, Elamri et al. [10] explained some of the impacts of shading conditions in Agrivoltaic on the development of the root system and root water uptake. Lettuce root profiles measured 13 days after transplanting exhibited rooting depths of approximately 15 cm, with root biomass closely corresponding to total fresh biomass. On the other hand, Kostik et al. [19] suggested that Agrivoltaic conditions should be preferred for plants with low root density and high shade tolerance.

**Figure 7:**
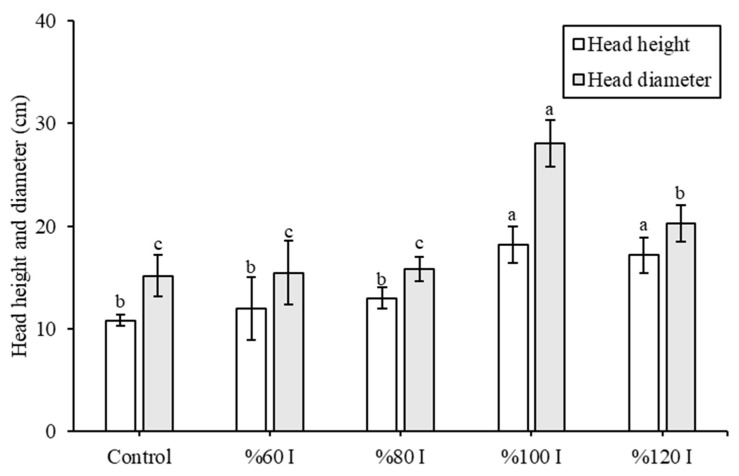
Head sizes of lettuce plants grown under different irrigation treatments in the Agrivoltaic and the control (open field). The different letter indicates a significant difference (*p* < 0.05).

According to Figure 7, the %100 I and %120 I groups exhibited significantly the highest mean head height (*p* < 0.05). In the control group, head height was found to be 10.83 ± 0.58 cm lower compared to Agrivoltaic conditions of %60 I (12.00 ± 3.04), %80 I (13.00 ± 1.00), %100 I (18.17 ± 1.76), and %120 I (17.17 ± 1.76). The %100 I group also had the largest mean head diameter, measuring 28.03 ± 2.24 cm (*p* < 0.05). Light intensity and temperature directly influence lettuce leaf growth, but effects may be overcome by adequate and well-timed irrigation. Such conditions may help maintain leaf turgor, support photosynthesis, and prevent overheating through evaporative cooling. Our findings are consistent with those of Zheng et al. [20], who compared the average maximal width of lettuce before harvest (mm) under the natural state (130.89), conventional Agrivoltaic system (121.89), even-lighting Agrivoltaic system (137.33), and even-lighting Agrivoltaic system with LED lamp (128.85) growing conditions. Compared to Marrau et al. [7], both broader and longer lettuce leaves under Agrivoltaic conditions compared to those in the full-sun control group were reported, which aligns with our findings under Agrivoltaic.

**Figure 8:**
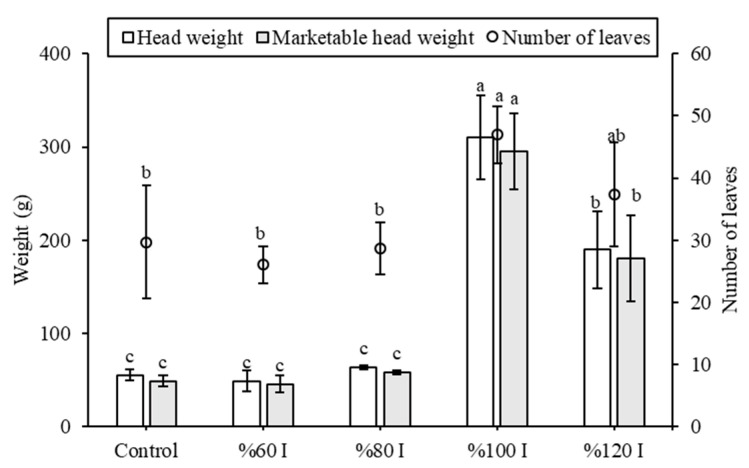
Head properties of lettuce plants grown under different irrigation treatments in the Agrivoltaic and the control (open field). The different letter indicates a significant difference (*p* < 0.05).

In terms of head weight, marketable head weight, and the number of leaves, the %100 I group exhibited the highest values, recorded as 310.05 ± 44.80 g, 295.03 ± 40.99 g, and 47 ± 4.58, respectively. These results were followed by the %120 I group (Figure 8). No statistically significant differences were observed among the control, %60 I, and %80 I groups (*p* < 0.05). Under low light or cool temperatures, lettuce has lower water demand, and excessive irrigation may also cause waterlogging, reduce oxygen availability to roots, and weaken leaf development. Therefore, proper irrigation practices—adjusted to light intensity and temperature—are essential to ensure healthy and high-quality lettuce leaves. Similarly, Zheng et al. [20] determined the average leaf number before the harvest and average fresh weight (g) of lettuce grown under the natural state (16.00, 17.89 g), conventional Agrivoltaic system (12.09, 8.32 g), even-lighting Agrivoltaic system (14.69, 18.44 g), and even-lighting Agrivoltaic system with LED lamps (14.36, 18.32 g); our results confirm these benefits while also demonstrating in soil conditions instead of planting pots. Unlike Jamil et al. [21], who focused on the transparencies of solar panels for Agrivoltaic in a controlled environment room (recreated temperatures typical of an outdoor summer growing season in London, ON, Canada), our study shows that plants under agrivoltaic can achieve higher leaf counts than the control group. These results highlight the significant influence of climate conditions.

### 2.4. Leaf Color Characteristics

In Table 1, the color parameters (L*, a*, b*, C, α, and ∆E) of the control group and the %60 I, %80 I, %100 I, and %120 I groups were compared. The highest L* value (50.29 ± 2.69) was recorded in the %120 I group, while the highest—a* (greenness) value (−10.39 ± 0.85) was observed in the %100 I group (*p* < 0.05). On the other hand, the lowest α value (107.18 ± 1.99) was recorded in the %120 I group. Furthermore, no statistically significant differences were found among the groups for b, C, and ∆E values (*p* < 0.05). Fontana et al. [22] conducted a color analysis on lettuce leaves grown under different cultivation conditions (conventional, hydroponic, and organic). The L, a, b, α, and C values of the three lettuce samples they analyzed were 65.3, 67.7, and 60.6; −14.6, −17.3, and −16.5; 31.8, 36.1, and 34.3; 114.7, 115.6, and 115.7; and 34.9, 40.0, and 38.1, respectively. Their findings align with the results obtained under the Agrivoltaic conditions applied in this study.

## 3. Materials and Methods

### 3.1. Experimental Site and Setup Description

**Figure 9:**
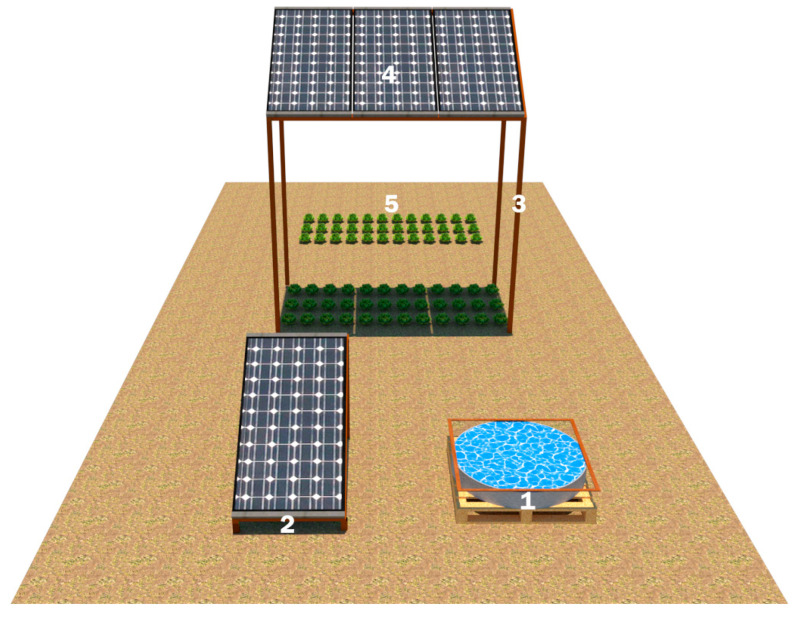
Experimental setup.

The experiments were conducted at the Agricultural Research and Application Field of the Faculty of Agriculture (40°13′49″ N, 28°51′38″ E). The experimental setup is shown in Figure 9. In front, an A-class evaporation pan (1) to measure irrigation requirements and a control (ground-mounted) solar panel (2) (south-facing with an angle of approximately 25°) were installed. In the middle, the supporting system was constructed by shaping tree branches (3), which are pruning residues from the forests owned by Bursa Uludağ University. The supporting system was designed with the front legs positioned at a height of 185 cm from the ground and the rear legs at a height of 245 cm. The distance between the legs is 130 × 290 cm (3.77 m^2^), and the Agrivoltaic system’s PV panels (4) (SFP250, Solarfield, Türkiye) are also positioned south at an angle of approximately 25°. Behind, the control group (3.77 m^2^), lettuce was grown (5). The plant material used was the curly lettuce (*Lactuca sativa* L.) variety of “sürpriz”. The variety is characterized by late bolting, medium green leaf color, thick leaves, juiciness, crispness, good taste, and a high number of leaves. It is also defined as suitable for open-field cultivation during the summer months [23]. During the study, climatic measurements such as temperature, humidity, wind speed, wind direction (Cem, DT-618, Shenzhen, China), radiation (Cem, DT-1307, Shenzhen, China), and light intensity (TT Technic, VC1010A, Shenzhen, China) were conducted at 13:00.

### 3.2. Test Procedure

In the study, the seedling was purchased from Agromar Seedling Company, located in Bursa, Türkiye. Homogeneous (healthy and uniform) selections were made within the seedlings before planting. Lettuce seedlings were planted on 6 August 2024, with a row spacing of 0.50 m and an in-row spacing of 0.25 m. Throughout the experimental period, no pesticide or fertilizer applications were conducted. The harvest was carried out on 8 October 2024.

Four different irrigation treatments were tested by calculating evaporation from the A-class evaporation pan over a specific time period using different pan coefficients. A total of four experimental treatments (60%, 80%, 100% and 120%) were conducted in a randomized block design with three replications. Additionally, the control group was conducted outside the Agrivoltaic area with an irrigation rate of 100%.

The amounts of water applied to the plots based on the research treatments were calculated using the following equation, considering the evaporation values from the A-class evaporation pan [24].

(1) $$I=E_{pan}\times K_{pc}\times A$$

Here

*I*: Irrigation water amount (liters/plant);
*E_pan_*: Evaporation amount from the evaporation pan (mm);
*K_pc_*: Evaporation pan coefficient;
*A*: Area per plant (m^2^/plant).

Based on morphological and physicochemical analysis, the soil was classified as Eutric Vertisol according to the Soil Taxonomy and FAO/UNESCO classification systems. The soil pH was slightly acidic in surface horizons but slightly alkaline in sub horizons due to the increase in CaCO_3_ content (pH = 6.50–7.84). Organic matter contents were low and diminishing by depth (% Org. M. = 1.51–0.18) [25].

### 3.3. Measurements of Leaf Temperature, SPAD, and Panel Power

During the cultivation period, leaf temperature and total SPAD readings were recorded from both the control and 100% I groups under the Agrivoltaic system, using an infrared thermometer (Fluke, 62 Max+, Washington, DC, USA) [26] and a chlorophyll meter (Konica Minolta, SPAD 502 Plus, Osaka, Japan) [27], respectively. On the other hand, the power output of PV panels operating under control (ground-mounted) and agrivoltaic conditions (above) was determined using a solar panel analyzer (Prova, 210, New Taipei City, Taiwan) [28].

### 3.4. Determination of the Size of Root and Plant, Plant Weight, and Number of Leaves

At the time of harvest, root length and width were determined from the root collar following careful extraction and washing of the roots to prevent mechanical damage. The root collar diameter was measured at two points using a digital caliper. Furthermore, head diameters were obtained using a ruler, while head lengths were assessed by measuring from the root collar region to the tip of the outermost leaves. Moreover, head weights were achieved by weighing the lettuce heads on a precision scale after the roots were removed. The marketable head weight was scaled by reweighing the heads after removing low-quality leaves. Additionally, the number of leaves was counted per plant from the outermost to the innermost [24,27].

### 3.5. Leaf Color Analysis

In lettuce plants, color measurements were conducted on the third leaf from the outside to the inside using a colorimeter device (Hunterlab, MSEZ-4500L, Reston, VA, USA), recording L*, a*, and b* values. L* represents lightness or darkness, a* indicates the balance between green (−) and red (+), and b* represents the balance between blue (−) and yellow (+). The control group samples were designated as reference values, expressed as L_0_*, a_0_*, and b_0_*. To evaluate color changes, Equations (2)–(4) were used to calculate the color saturation (C), hue angle (α), and total color difference (ΔE), respectively [22].

(2) $$C=\sqrt{\left(a^{2}+b^{2}\right)}$$

(3) $$\alpha =\tan^{-1}(\frac{b}{a})$$

(4) $$\Delta E=\sqrt{{\left(L^{*}-L_{0}^{*}\right)}^{2}+{\left(a^{*}-a_{0}^{*}\right)}^{2}+{\left(b^{*}-b_{0}^{*}\right)}^{2}}$$

### 3.6. Statictics

The data were analyzed using the statistical software JMP (Version 7.0, SAS Institute Inc., Cary, NC, USA), and variance analysis was performed using one-way ANOVA. To determine significant differences among the samples, the Least Significant Difference (LSD) test was applied at a 95% confidence level (*p* < 0.05).

## 4. Conclusions

This study investigated the performance of an Agrivoltaic system constructed from unprocessed pruning residues, evaluating its impact on some lettuce (*Lactuca sativa* L.) parameters and PV power generation. With the use of locally sourced natural material for the support structure, it is aimed that the environmental footprint of Agrivoltaic installations is reduced, while promoting sustainable land use. Among the irrigation treatments, the 100% irrigation level under Agrivoltaic conditions showed the most favorable outcomes in terms of head weight, marketable head weight, leaf numbers, root parameters, and green leaf development. These findings underline the importance of optimized water management in shaded environments. Despite the slightly reduced power output compared to ground-mounted PV systems, the Agrivoltaic system still demonstrated adequate energy generation, highlighting the viability of dual-use land systems for both agricultural and energy purposes. Additionally, the system moderated leaf temperature and preserved soil moisture, indicating potential benefits for crop resilience in regions prone to high solar radiation and water scarcity.

On the other hand, the integrated nature of Agrivoltaic systems introduces certain complexities. The effects of altered microclimatic variables, such as light intensity, temperature, and humidity, present a methodological limitation. Moreover, the long-term durability and structural performance of support systems made from pruning residues remain uncertain and require further material testing under real environmental conditions. Therefore, future research should focus on disentangling the individual contributions of microclimatic factors through controlled experiments or simulation models. Additionally, exploring dynamic or adjustable panel systems, integrating smart irrigation technologies, and assessing crop-specific responses across various climates and soil types would enhance the broader applicability of Agrivoltaics. In conclusion, Agrivoltaic systems constructed with bio-based materials offer a promising pathway for sustainable food-energy integration.

## Figures and Tables

**Table 1:** Color parameters (lightness (L*, +), green (a*, −), yellow (b*, +). color saturation (C), hue angle (α), and total color difference (ΔE) of lettuce leaves grown under different irrigation treatments in the Agrivoltaic and the control (open field).

	L*	a*	b*	C	α	∆E
Control	49.09 ± 1.97 ab	−8.87 ± 0.53 ab	27.04 ± 2.76 a	28.46 ± 2.78 a	108.20 ± 0.70 ab	-
%60 I	47.26 ± 2.12 ab	−8.45 ± 0.62 a	27.52 ± 3.25 a	28.80 ± 3.15 a	107.18 ± 1.99 b	5.35 ± 1.13 a
%80 I	46.24 ± 2.63 b	−8.99 ± 0.79 ab	24.64 ± 4.37 a	26.23 ± 4.36 a	110.25 ± 1.87 a	6.30 ± 0.65 a
%100 I	47.21 ± 1.42 ab	−10.39 ± 0.85 c	28.79 ± 2.75 a	30.61 ± 2.87 a	109.87 ± 0.45 a	6.40 ± 1.11 a
%120 I	50.29 ± 2.69 a	−9.87 ± 0.86 bc	28.06 ± 3.22 a	29.75 ± 3.32 a	109.42 ± 0.53 ab	6.69 ± 2.47 a

The different letter indicates a significant difference (*p* < 0.05).

## Data Availability

Data are contained within the article.
